# Impact of maternal neuraxial labor analgesia exposure on offspring's neurodevelopment: A longitudinal prospective cohort study with propensity score matching

**DOI:** 10.3389/fpubh.2022.831538

**Published:** 2022-07-29

**Authors:** Chun-Mei Deng, Ting Ding, Zhi-Hua Liu, Shu-Ting He, Jia-Hui Ma, Ming-Jun Xu, Lei Wang, Ming Li, Wei-Lan Liang, Xue-Ying Li, Daqing Ma, Dong-Xin Wang

**Affiliations:** ^1^Department of Anesthesiology and Critical Care Medicine, Peking University First Hospital, Beijing, China; ^2^Department of Anesthesiology, Beijing Obstetrics and Gynecology Hospital, Beijing, China; ^3^Department of Anesthesiology, Haidian Maternal & Child Health Hospital, Beijing, China; ^4^Department of Pediatrics, Peking University First Hospital, Xicheng District, Beijing, China; ^5^Department of Biostatistics, Peking University First Hospital, Beijing, China; ^6^Section of Anesthetics, Pain Management and Intensive Care, Department of Surgery and Cancer, Imperial College London, Chelsea and Westminster Hospital, London, United Kingdom; ^7^Outcomes Research Consortium, Cleveland, OH, United States

**Keywords:** labor, obstetric [MeSH], analgesia obstetric, depression postpartum, child development, cognition

## Abstract

**Background:**

Neuraxial analgesia is widely used to relieve labor pain; its effects on long-term neurodevelopment of offspring remain unclear. This study was designed to investigate the influence of maternal neuraxial labor analgesia on offspring mental development.

**Methods:**

This was a predefined secondary analysis of a 2-year prospective longitudinal study. Nulliparous women with single-term cephalic pregnancy preparing for vaginal delivery self-selected neuraxial analgesia or not during labor. Mothers and their offspring were followed up 2 years later. children's mental development was assessed with the bayley scales of infant development. A multivariable logistic model was used to identify factors associated with below-average mental development (Mental Development Index <90).

**Results:**

A Total of 508 pairs of mothers and children completed a 2-year follow-up. after propensity score matching, 387 pairs were included in the analysis. In both cohorts, the proportions with below-average mental development were slightly lower in children whose mothers received neuraxial labor analgesia, although not statistically significant [in the full cohort: 9.8 % (36/368) vs. 15.7% (22/140), *P* = 0.060; In the matched cohort: 8.3% (21/254) vs. 14.3% (19/133), *P* = 0.065]. A higher 2-year depression score (in the full cohort: Odds Ratio 1.15, 95% CI 1.08–1.22, *P* < 0.001; In the matched cohort: Odds Ratio 1.09, 95% CI 1.01–1.18, *P* = 0.037), but not neuraxial analgesia exposure, was associated with an increased risk of below-average mental development.

**Conclusions:**

Maternal depression at 2 years was associated with the risk of below-average mental development, whereas maternal exposure to neuraxial labor analgesia was not.

**Clinical Trial Registration:**

The study was registered with www.chictr.org.cn (ChiCTR-OCH-14004888) and ClinicalTrials.gov (NCT02823418).

## Introduction

How perinatal factors affect neurodevelopment of children has always been an issue of much concern. Prenatal maternal stress has been reported to be associated with impaired cognitive development, poor intellectual performance, as well as behavioral and emotional problems during childhood (1, 2). As one of the most painful events during a woman's lifetime, the intense labor pain provokes serious maternal stress responses, which are related to impaired uterine contraction (3), neonatal hypoxia and metabolic acidosis (4), and even maternal postpartum depression (5). It is also possible that the maternal and fetal stress during labor may produce long-lasting effects on children (6), which has been verified in animal models (7). Indeed, numerous studies suggest that postpartum depression may affect long-term physical and neurodevelopment of children (8, 9).

Neuraxial labor analgesia, including epidural analgesia and combined spinal-epidural analgesia, is a well-established technique to relieve labor pain. It can help to reduce the maternal stress response during labor (10) and might be associated with a lower risk of maternal postpartum depression (11, 12); all these may be beneficial to the long-term development in offspring. On the other hand, neuraxial labor analgesia increases intrapartum maternal fever and instrumental delivery, which may potentially worsen neonatal outcomes and increase birth trauma (13, 14). In addition, anesthetic exposure during neuraxial analgesia may lead to fetal-neonatal depression (15) and even neurotoxic effects of less mature neonatal brain (16). Taking all these into account, the potential long-term effects of neuraxial analgesia on offspring neurodevelopment are still controversial and deserve further study. The objective of this analysis was to investigate if there is any association between maternal exposure to neuraxial analgesia during labor and neurocognitive development in offspring at 2 years of age.

## Materials and methods

### Study design

This was a predefined secondary analysis of a 2-year prospective longitudinal study. The study was conducted in Peking University First Hospital (a tertiary general hospital), Beijing Obstetrics and Gynecology Hospital (a tertiary specialized hospital) and Haidian Maternal & Child Health Hospital (a secondary specialized hospital) in Beijing, China. Results of the underlying study have been published elsewhere (11, 17).

### Participant recruitment

We enrolled nulliparae with a single-term cephalic pregnancy preparing for vaginal delivery. Women who met any of the following criteria were excluded: age <18 years or >34 years; a history of psychiatric disease (schizophrenia); contraindications to neuraxial analgesia, such as diseases of the central nervous system (e.g., poliomyelitis, cerebrospinal meningitis, encephalitis), spinal or intraspinal diseases (e.g., trauma or surgery of spinal column, intraspinal canal mass, lumbar disc herniation), systematic infectious diseases (e.g., sepsis, bacteremia), infection of skin or soft tissue at the site of puncture, and coagulopathy; or delivery room admission outside daytime working hours.

### Conduct of neuraxial labor analgesia

After being informed about the benefits and potential risks of neuraxial labor analgesia, parturients self-decided to receive neuraxial labor analgesia or not. Epidural analgesia or combined spinal-epidural analgesia was performed for those who requested analgesia. Neuraxial analgesia was initiated when the cervix was dilated to 1 cm or more. A patient-controlled epidural analgesia (PCEA) pump established with a mixture of 0.1% ropivacaine plus 0.5 μg/ml sufentanil was attached to the epidural catheter to maintain analgesia until end of the third stage of labor. Routine care was provided for those who did not request neuraxial analgesia, including intramuscular meperidine prescribed by the obstetricians. Detailed procedures of neuraxial analgesia were described previously (11, 17).

### Baseline and perinatal data collection

Baseline data of mothers and fathers were collected. Prenatal assessments were completed by parturients themselves at admission to the delivery ward. Depressive symptoms were assessed with the Edinburgh Postnatal Depression Scale [EPDS, a 10-item self-report questionnaire; the total score ranges from 0 to 30, with higher score indicating more severe depressive symptoms (18)]. Marriage satisfaction was assessed with the ENRICH Marital Satisfaction Scale [total score ranges from 10 to 50, with higher score indicating better marital satisfaction (19)]. Anxiety level was assessed with the Zung Self-Rating Anxiety Scale [total score ranges from 25 to 100, with higher score indicating more severe anxiety (20)]. Social support was assessed with the Social Support Rating Scale [total score ranges from 11 to 62, with higher score indicating better social support (21)]. The Chinese versions of the above instruments had been validated (22–25).

Intrapartum data included use of neuraxial analgesia, duration of labor, the highest body temperature, mode of delivery, lateral episiotomy, and estimated blood loss. Neonatal data included sex, birth weight, Apgar scores at 1 and 5 min after birth, and postnatal management (admission to neonatal ward or intensive care unit). A telephone follow-up was performed at 6 weeks (42–49 days) postpartum. The severity of maternal depression was assessed with the EPDS; and an EPDS score ≥10 was defined as the threshold of postpartum depression (25). The condition of neonates, the mode of baby feeding, the existence of persistent pain and the NRS pain score were recorded.

### Two-year follow-up of mothers and children

A face-to-face interview was completed between 23 and 24 months after childbirth. Maternal data were collected and included body weight and height, duration of breast-feeding, new-onset diseases requiring therapy or surgical procedures, and another childbirth. Social support was assessed with the Social Support Rating Scale. Depressive symptoms were assessed with the EPDS, and those with a 2-year EPDS score ≥10 were defined as having 2-year depression (25). Infant data was collected and included month age, body weight and height, time to start complementary feeding, pediatric diseases requiring therapy or surgical procedures. Physical development was evaluated according to the Reference Standard for Growth and Development of Children under 7 Years of Age in China which was released by the Ministry of Health of China on 2 June 2009 (26). A height or weight of < -2 standard deviation (SD) was defined as physical development delay.

Neurocognitive development of infants was assessed with the Chinese Revision of Bayley Scales of Infant Development, which has been validated in Chinese urban children aged from 2 to 30 months (27) and widely used in related studies (28). It has two primary subtests, i.e., the mental scale, which includes 163 items and evaluates children's cognition, language and social development, and the psychomotor scale, which includes 81 items and assesses gross and fine motor development. The Mental Development Index (MDI) and Psychomotor Development Index (PDI) were converted from the age-adjusted raw scores of mental and psychomotor scales, respectively. The average scores of MDI and PDI in normal urban children are both 100 with a SD of 15, with higher scores indicating better neurocognitive development. The MDI and PDI scores were classified into seven levels, i.e., developmental delay (<70), borderline (70–79), below average (80–89), middle level (90–109), above average (110–119), good (120–129), and outstanding (≥130) (27). In the present study, a MDI score <90 was defined as below-average mental development and a PDI <90 as below-average psychomotor development. Investigators who performed neurocognitive assessment were trained to use the Bayley Scales of Infant Development and were blinded to the exposure to neuraxial analgesia during labor. The primary endpoint was occurrence of below-average mental development at 2 years of age.

### Statistical analysis

Mothers and their offspring were divided into two groups according to neuraxial analgesia exposure during labor. Between-group differences of baseline variables were compared using the absolute standardized differences (ASDs), which are defined as the absolute difference in means, mean ranks or proportions divided by the pooled standard deviation and calculated with the formula published by Austin (29). An ASD ≥ 0.195 (i.e., 1.96$\times \sqrt{\left(n1\;+\;n2\right)/\left(n1\;\times \;n2\right)\;}$) was considered unbalanced between the two groups. For intrapartum and postpartum variables, continuous variables were compared with independent samples *t*-test or Mann-Whitney *U* test, and categorical variables were compared with χ2 test or Fisher's exact test.

Baseline variables that were considered clinically relevant were used for propensity score matching in order to balance the potential bias in selecting neuraxial analgesia. These variables were selected a priori and included sociodemographic characteristics, medical history before last pregnancy, history of last pregnancy, prenatal hemoglobin, as well as prenatal assessment results of depression, marital satisfaction, anxiety, and social support (30, 31). A logistic regression model was used to calculate propensity scores predicting the probability of receiving neuraxial labor analgesia. In the present study, we carried out a 1:2 matching without replacement using the nearest-neighbor matching algorithm with caliper widths equal to 0.2.

For both the full cohort and the matched cohort, univariable logistic regression analyses were performed to screen variables that might be associated with below-average mental development in 2-year-old offspring. After testing for collinearity, factors with *P* < 0.15 in univariable analyses or were considered clinically important were included in a multivariable logistic regression model to identify independent factors associated with below-average mental development using a backward procedure. To further explore the impact of neuraxial analgesia duration on children's neurocognitive development, we divided neuraxial analgesia exposure time into 4 levels, i.e., no analgesia, <4 h, 4 to 8 h, and more than 8 h, and adjusted with the same aforementioned covariates in multivariable logistic regression models. An exploratory analysis was performed to further clarify the association between neuraxial analgesia and cognitive development delay in 2-year-old offspring with the MDI cutoff score set at 80. A two-tailed *P* value of <0.05 was considered statistically significant. Statistical analyses were performed with the SPSS 25.0 software (IBM SPSS Inc., Chicago, IL, USA) and the free software package “R” version 2.15.3 including the “Matchit” and the “ROC” plugin.

## Results

### Participants

**Figure 1:**
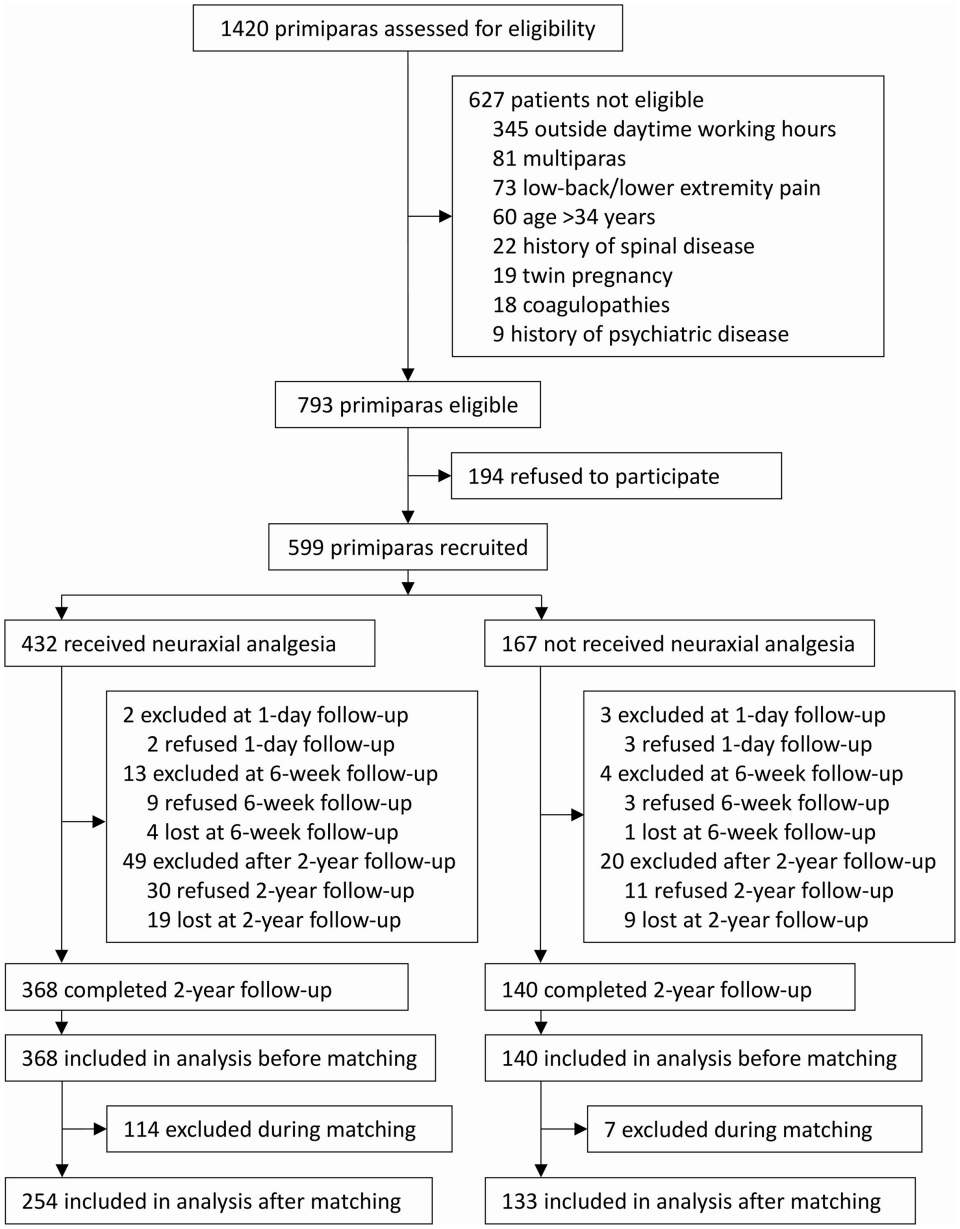
Flow chart of the study.

From 1 August 2014 to 29 May 2015, 599 nulliparae were enrolled after obtaining written informed consents. Among these, 577 mothers and their neonates completed the 1-day and 6-week follow-ups (17 refused and 5 were lost to follow-up). From 9 July 2016 to 25 April 2017, 508 mothers and their offspring completed the 2-year follow-up (41 refused follow-up and 28 were lost to follow-up) and were included in the final analysis. There were no significant differences regarding demographic and baseline data between parturients who completed the 2-year followed-up and those who did not (Supplementary Table S1). Of the included 508 mothers, 368 (72.4%) were given neuraxial analgesia and 140 (27.6%) were not. After propensity score matching, 387 mothers and their offspring remained in the analysis, of whom 254 mothers (65.6%) were given neuraxial analgesia and 133 (34.4%) were not (Figure 1).

### Baseline and perinatal data

Of the 508 mothers who completed the study, the proportions of attending childbirth classes and husband with stable occupation were higher, whereas the proportion of covered by social health insurance was lower in those who received neuraxial analgesia than in those who did not. Twenty-four variables collected at the baseline were used for propensity score matching. In the matched cohort, all baseline variables were well balanced (Table 1).

**Table 1:** Baseline data used for propensity score matching.

		**Full cohort**			**Matched cohort**		
	**Total** **(*****n*** = **508)**	**Neuraxial analgesia** **(*****n** **=*** **368)**	**No neuraxial analgesia** **(*****n** **=*** **140)**	**ASD**	**Neuraxial analgesia** **(*****n** **=*** **254)**	**No neuraxial analgesia** **(*****n** **=*** **133)**	**ASD**
**Maternal data**							
Age at childbirth (year)	30 (28–32)	30 (28–32)	30 (28–32)	0.018	30 (28–32)	30 (28–32)	0.023
Antenatal body mass index (kg/m^2^)	27.3 ± 2.9	27.4 ± 2.7	27.0 ± 3.1	0.120	27.4 ± 2.7	27.1 ± 3.2	0.086
Han nationality^a^	479 (94.3%)	347 (94.3%)	132 (94.3%)	<0.001	240 (94.5%)	126 (94.7%)	0.011
With religion^b^	25 (4.9%)	18 (4.9%)	7 (5.0%)	0.005	14 (5.5%)	6 (4.5%)	0.048
Education >12 years	486 (95.7%)	350 (95.1%)	136 (97.1%)	0.122	246 (96.8%)	129 (97.0%)	0.008
Stable occupation	485 (95.5%)	354 (96.2%)	131 (93.6%)	0.107	242 (95.3%)	127 (95.5%)	0.010
Covered by social health insurance	480 (94.5%)	343 (93.2%)	137 (97.9%)	0.320	249 (98.0%)	130 (97.7%)	0.019
**Total family income (¥/month)**				0.104			0.004
<10,000	107 (21.1%)	80 (21.7%)	27 (19.3%)		53 (20.9%)	26 (19.5%)	
10,000–20,000	264 (52.0%)	186 (50.5%)	78 (55.7%)		132 (52.0%)	73 (54.9%)	
>20,000	137 (27.0%)	102 (27.7%)	35 (25.0%)		69 (27.2%)	34 (25.6%)	
**History before last pregnancy**							
Premenstrual syndrome^c^	49 (9.6%)	39 (10.6%)	10 (7.1%)	0.134	24 (9.4%)	10 (7.5%)	0.073
Medical comorbidity^d^	37 (7.3%)	26 (7.1%)	11 (7.9%)	0.029	18 (7.1%)	10 (7.5%)	0.016
Gynecological diseases^e^	51 (10.0%)	36 (9.8%)	15 (10.7%)	0.030	28 (11.0%)	14 (10.5%)	0.016
Previous surgery	76 (15.0%)	58 (15.8%)	18 (12.9%)	0.086	34 (13.4%)	18 (13.5%)	0.004
Adverse pregnancy history^f^	171 (33.7%)	116 (31.5%)	55 (39.3%)	0.158	98 (38.6%)	49 (36.8%)	0.036
**History of last pregnancy**							
Duration of pregnancy (day)	279 (273–281)	279 (273–281)	280 (273–282)	0.093	280 (273–282)	279 (273–282)	0.029
Smoking/drinking during pregnancy	4 (0.8%)	2 (0.5%)	2 (1.4%)	0.074	0 (0%)	1 (0.8%)	0.087
Stressful life events^g^	52 (10.2%)	39 (10.6%)	13 (9.3%)	0.045	25 (9.8%)	13 (9.8%)	0.002
Attend childbirth classes	409 (80.5%)	306 (83.2%)	103 (73.6%)	0.216	199 (78.3%)	101 (75.8%)	0.056
**Obstetric diseases**	159 (31.3%)	116 (31.5%)	43 (30.7%)	0.017	80 (31.5%)	42 (31.6%)	0.002
Gestational diabetes mellitus	115 (22.6%)	86 (23.4%)	29 (20.7%)	0.065	60 (23.6%)	28 (21.1%)	0.063
Pregnancy-induced hypertension	28 (5.5%)	21 (5.7%)	7 (5.0%)	0.032	14 (5.5%)	7 (5.3%)	0.011
Hypothyroidism	40 (7.9%)	30 (8.2%)	10 (7.1%)	0.039	21 (8.3%)	10 (7.5%)	0.028
Prepartum hemoglobin (g/L)	12.4 ± 1.2	12.5 ± 1.2	12.4 ± 1.1	0.012	12.4 ± 1.2	12.5 ± 1.1	0.018
Edinburgh postnatal depression scale (score)	7 (5–8)	6 (5–8)	7 (5–8)	0.064	6 (5–8)	7 (5–8)	0.078
ENRICH marital satisfaction scale (score)	47 (45–49)	47 (45–48)	48 (46–49)	0.073	47 (45–49)	48 (46–49)	0.028
Zung self-rating anxiety scale (score)	34 (31–38)	35 (31–38)	34 (31–38)	0.115	34 (30–38)	34 (31–37)	0.091
Social support rating scale (score)	40 (38–43)	40 (38–43)	40 (37–43)	0.053	40 (38–43)	40 (37–43)	0.075
**Paternal data**							
Education of husband >12 years	488 (96.1%)	354 (96.2%)	134 (95.7%)	0.025	246 (96.9%)	128 (96.2%)	0.032
With stable occupation	504 (99.2%)	367 (99.7%)	137 (97.9%)	0.359	253 (99.6%)	131 (98.5%)	0.091

Data are presented as mean ± SD, n (%), or median (interquartile range). ASD values in bold indicate those ≥0.195.

ASD, absolute standardized difference (an ASD of ≥0.195 is considered imbalanced between the two groups); ¥, Chinese yuan.

a Other nationalities include Manchu, Mongol, Huis, Koreans, and Yi.

b Include Buddhism, Islam, and Christianism.

c Refers to symptoms of irritability, fatigue, depression and headache that repeatedly occurred during the luteal phase of the menstrual cycle and affected daily life. Diagnosed by the gynecologists.

d Include asthma, arrhythmia, thyroid disease, nephritis, nephritic syndrome, and positive hepatitis B surface antigen.

e Include uterine fibroid, ovarian cyst, endometriosis, polycystic ovary syndrome, and primary amenorrhea.

f Include arrest of fetal development, spontaneous abortion, and induced abortion.

g Include bereavement, accidental injury, layoff or unemployment.

In the full cohort, mothers with neuraxial labor analgesia suffered from more intrapartum fever (≥37.5 °C), had longer durations of the first and second labor stages, underwent less cesarean delivery (but gave more spontaneous and instrumental delivery), and experienced less postpartum depression at 6 weeks when compared with those without (Table 2). In the matched cohort, the above differences were also present between the two groups (Table 2).

**Table 2:** Intra- and postpartum data before and after matching.

		**Full cohort**			**Matched cohort**		
	**Total** **(*****n*** = **508)**	**Neuraxial analgesia** **(*****n*** **=** **368)**	**No neuraxial analgesia** **(*****n*** **=** **140)**	***P*** **-value**	**Neuraxial analgesia** **(*****n*** **=** **254)**	**No neuraxial analgesia** **(*****n*** **=** **133)**	***P*** **-value**
**Maternal data**							
**Highest temperature during labor**							
≥37.5°C	65 (12.8%)	57 (15.5%)	8 (5.7%)	0.003	39 (15.4%)	8 (6.0%)	0.008
≥38.0°C	10 (2.0%)	9 (2.4%)	1 (0.7%)	0.368	6 (2.4%)	1 (0.8%)	0.430
**Duration of labor^a^**							
First stage (min)	540 (350–780)	600 (420–840)	318 (221–540)	<0.001	600 (420–860)	320 (223–540)	<0.001
Second stage (min)	46 (28–79)	51 (32–83)	34 (20–56)	<0.001	51 (31–83)	35 (21–56)	<0.001
Third stage (min)	7 (5–10)	7 (5–10)	8 (4–10)	0.887	7 (5–10)	8 (5–10)	0.737
Use of oxytocin	344 (67.7%)	243 (66.0%)	101 (72.1%)	0.188	171 (67.3%)	97 (72.9%)	0.256
Artificial membrane rupture	195(38.4%)	146 (39.7%)	49 (35.0%)	0.333	103 (40.6%)	47 (35.3%)	0.317
**Mode of delivery**				<0.001			<0.001
Spontaneous delivery	336 (66.1%)	255 (69.3%)	81 (57.9%)		180 (70.9%)	76 (57.1%)	
Forceps delivery	49 (9.6%)	42 (11.4%)	7 (5.0%)		27 (10.6%)	6 (4.5%)	
Cesarean delivery	123 (24.2%)	71 (19.3%)	52 (37.1%)		47 (18.5%)	51 (38.3%)	
Estimated blood loss (ml)	200 (150–300)	200 (150–348)	260 (200–300)	0.810	200 (150–350)	260 (200–300)	0.883
**Neonatal data**							
Male sex	274 (53.9%)	205 (55.7%)	69 (49.3%)	0.195	143 (56.3%)	64 (48.1%)	0.126
Birth weight (g)	3,416 ± 405	3,429 ± 399	3,383 ± 420	0.256	3,444 ± 407	3,392 ± 427	0.237
**Apgar score after birth (score)**							
1 min	10 (10–10)	10 (10–10)	10 (10–10)	0.976	10 (10–10)	10 (10–10)	0.936
5 min	10 (10–10)	10 (10–10)	10 (10–10)	0.547	10 (10–10)	10 (10–10)	0.497
Admission to neonatal ward^b^	48 (9.4%)	36 (9.8%)	12 (8.6%)	0.677	28 (11.0%)	12 (9.0%)	0.539
**6-week postpartum data**							
Exclusive breast feeding	351 (69.1%)	257 (69.8%)	94 (67.1%)	0.557	178 (70.1%)	88 (66.2%)	0.430
**Persistent pain^c^**	117 (23.0%)	90 (24.5%)	27 (19.3%)	0.216	63 (24.8%)	25 (18.8%)	0.181
Numeric rating scale of pain ≥4	63 (12.4%)	49 (13.3%)	14 (10.0%)	0.311	34 (13.4%)	13 (9.8%)	0.302
Edinburgh postnatal depression scale (score)	6 (4–9)	6 (4–9)	6 (4–10)	0.077	6 (4–8)	6 (4–10)	0.084
Postpartum depression^d^	90 (17.7%)	53 (14.4%)	37 (26.4%)	0.002	33 (13.0%)	34 (25.6%)	0.002

Data are mean ± SD, n (%), or median (interquartile range). P values in bold indicate those < 0.05.

a Exclude those who underwent Cesarean delivery.

b Neonates were admitted to neonatal ward because of fetal distress/asphyxia, aspiration pneumonia, premature birth/low-birth weight, glucopenia, jaundice/hyperbilirubinemia, infection, convulsion, and anal atresia.

c Defined as persistent or recurrent pain that lasted for more than 1 months after childbirth.

d Defined as Edinburgh Postnatal Depression Scale ≥ 10.

### Outcomes of 2-year follow-up

In the full cohort, mothers with neuraxial labor analgesia had a lower 2-year EPDS score [median 3 (IQR 1–4) vs. 3 (2–6), *P* = 0.017] and a lower prevalence of 2-year depression [7.3% (27/368) vs. 13.6% (19/140), *P* = 0.029] when compared with those without. The offspring of mothers with neuraxial analgesia had a later start of complementary feeding [6 months (5–6) vs. 5 months (5–6), *P* = 0.010] and a higher Mental Development Index score at 2 years [111 (102–115) vs. 111 (102–114), *P* = 0.030] when compared with those of mothers without; they had a slightly lower rate of below-average MDI [9.8% (36/368) vs. 15.7% (22/140), *P* = 0.060] but not statistically significantly (Table 3, Supplementary Table S2, Supplementary Figure S1).

**Table 3:** 2-year follow-up outcomes.

		**Full cohort**			**Matched cohort**		
	**Total** **(*****n*** = **508)**	**Neuraxial analgesia** **(*****n*** **=** **368)**	**No neuraxial analgesia** **(*****n*** **=** **140)**	***P*** **value**	**Neuraxial analgesia** **(*****n*** **=** **254)**	**No neuraxial analgesia** **(*****n*** **=** **133)**	***P*** **value**
**Mothers' outcomes**							
Body mass index (kg/m^2^)	21.7 ± 2.6	21.8 ± 2.5	21.7 ± 2.6	0.701	21.9 ± 2.5	21.8 ± 2.6	0.701
Maternal disease within 2 years^a^	37 (7.3%)	28 (7.6%)	9 (6.4%)	0.647	16 (6.3%)	8 (6.0%)	0.912
Maternal surgery within 2 years^b^	30 (5.9%)	25 (6.8%)	5 (3.6%)	0.169	17 (6.7%)	4 (3.0%)	0.129
Duration of breast-feeding (month)	13 (9–18)	13 (8–18)	13 (10–19)	0.276	12 (8–18)	13 (10–19)	0.312
Another childbirth	19 (3.7%)	14 (3.8%)	5 (3.6%)	0.902	10 (3.9%)	4 (3.0%)	0.779
Social support rating scale (score)	37(34–40)	37 (34–40)	37 (34–40)	0.470	36 (34–40)	37 (35–40)	0.203
2-year Edinburgh postnatal depression scale (score)	3 (1–5)	3 (1–4)	3 (2–6)	0.017	3 (1–4)	3 (2–6)	0.041
2-year depression ^c^	46 (9.1%)	27 (7.3%)	19 (13.6%)	0.029	15 (5.9%)	16 (12.0%)	0.035
**Children's outcomes**							
Height (cm)	90 (87–90)	90 (87–90)	90 (88–91)	0.183	90 (87–90)	90 (88–91)	0.484
Weight (kg)	12 (12–13)	12 (12–13)	12 (12–13)	0.685	12 (12–14)	12 (12–13)	0.586
Complementary feeding (month)	6 (5–6)	6 (5–6)	5 (5–6)	0.010	6 (5–6)	5 (5–6)	0.020
Physical development delay^d^	8 (1.6%)	7 (1.9%)	1 (0.7%)	0.335	6 (2.4%)	1 (0.8%)	0.430
Pediatric disease within 2 years^e^	52 (10.2%)	38 (10.3%)	14 (10.0%)	0.933	20 (7.9%)	12 (9.0%)	0.697
Pediatric surgery within 2 years^f^	2 (0.4%)	2 (0.5%)	0 (0.0%)	>0.999	2 (0.8%)	0 (0.0%)	0.548
Mental development index (score)	111 (102–115)	111 (102–115)	111 (102–114)	0.030	111 (102–115)	111 (102–114)	0.059
**Mental development level^g^**				0.127			0.127
<90	58 (11.4%)	36 (9.8%)	22 (15.7%)		21 (8.3%)	19 (14.3%)	
90–109	162 (31.9%)	116 (31.5%)	46 (32.9%)		77 (30.3%)	43 (32.3%)	
≥110	288 (56.7%)	216 (58.7%)	72 (51.4%)		156 (61.4%)	71 (53.4%)	
Below average mental development^h^	58 (11.4%)	36 (9.8%)	22 (15.7%)	0.060	21 (8.3%)	19 (14.3%)	0.065
Psychomotor Development Index (score)	107 (102–115)	107 (102–112)	107 (105–117)	0.113	107 (102–115)	107 (105–117)	0.275
**Psychomotor development level^g^**				0.883			0.776
<90	18 (3.5%)	13 (3.5%)	5 (3.6%)		11 (4.3%)	4 (3.0%)	
90–109	263 (51.8%)	193 (52.4%)	70 (50.0%)		124 (48.8%)	68 (51.1%)	
≥110	227 (44.7%)	162 (44.0%)	65 (46.4%)		119 (46.9%)	61 (45.9%)	
Below average psychomotor development^h^	18 (3.5%)	13 (3.5%)	5 (3.6%)	0.983	11 (4.3%)	4 (3.0%)	0.522

Data are mean ± SD, n (%) or median (interquartile range). P-values in bold indicate those < 0.05.

a Refers to new-onset diseases that occurred during the 2-year period after childbirth h and required therapy, including mammitis/mammary abscess, pelvic floor dysfunction, polycystic ovary syndrome, hypothyroidism, hyperthyroidism, Hashimoto's thyroiditis, thyroid cancer, cerebral infarction, IgA nephropathy, lumbar disc herniation, scoliosis and phalangeal fracture.

b Refers to any surgical procedures performed during the 2-year period after childbirth, including second Cesarean delivery, induced abortion, vaginal polypectomy, hysteromyomectomy, adnexectomy, incision and drainage of mammary abscess, cholecystectomy, thyroidectomy, and incision and internal fixation metatarsal fracture.

c Defined as Edinburgh Postnatal Depression Scale ≥10.

d Defined as height or weight < -2 standard deviation according to the reference standard (26).

e Includes any congenital (atrial septal defect, anal atresia and urachal fistula) and/or acquired diseases (bronchiolitis, febrile convulsion, Kawasaki disease, infant rash, eczema, urticarial, allergic dermatitis, pneumonia, anemia, inguinal hernia, and enteritis) that required therapy during the 2-year period.

f Includes any surgical procedures (inguinal herniorrhaphy and urachal fistula resection) performed during the 2-year period.

g Originally classified into seven levels according to the Mental or Psychomotor Development Index scores, i.e., level 1: developmental delay (<70), level 2: borderline (70–79), level 3: below average (80–89), level 4: middle level (90–109), level 5: above average (110–119), level 6: good (120–129), and level 7: outstanding (≥130).

h Defined as Mental Development Index or Psychomotor Development Index <90.

In the matched cohort, mothers with neuraxial labor analgesia had a lower 2-year EPDS score [3 (1–4) vs. 3 (2–6), *P* = 0.041] and a lower prevalence of 2-year depression [5.9% (15/254) vs. 12.0% (16/133), *P* = 0.035] when compared with those without. The offspring of mothers with neuraxial analgesia had a later start of complementary feeding [6 months (5–6) vs. 5 months (5–6), *P* = 0.020] when compared with those of mothers without; they had a slightly higher Mental Development Index score at 2 years [111 (102–115) vs. 111 (102–114), *P* = 0.059] and a slightly lower rate of below-average mental development [8.3% (21/254) vs. 14.3% (19/133), *P* = 0.065] but not statistically significant (Table 3, Supplementary Table S2).

### Factors associated with below-average mental development at 2 years of age

In both cohorts, six factors with *P* < 0.15 in univariable analyses (Supplementary Table S3) or considered clinically important were included in the multivariate logistic regression model. After correction for confounding factors, a higher 2-year EPDS score of mothers was the only independent factor that significantly associated with an increased risk of below-average mental development in their 2-year-old offspring (in full cohort: OR 1.15, 95% CI 1.08–1.22, *P* < 0.001; in matched cohort: OR 1.09, 95% CI 1.01–1.18, *P* = 0.037; Table 4), whereas maternal exposure to neuraxial analgesia during labor or duration of neuraxial analgesia was not (Supplementary Table S4). Similar results were observed when the cutoff point of MDI was set at 80, i.e., there was no significant association between maternal exposure of neuraxial labor analgesia and cognitive development delay in 2-year-old offspring (Supplementary Table S5).

**Table 4:** Factors associated with below-average mental development in 2-year-old children^a^.

**Variables**	**Full cohort**			**Matched cohort**		
	**Univariate analysis**	**Multivariate analysis** ^**b**^		**Univariate analysis**	**Multivariate analysis** ^**c**^	
	***P*** **-value**	**Odds ratio (95% CI)**	***P*** **-value**	***P*** **-value**	**Odds ratio (95% CI)**	***P*** **-value**
Antenatal stressful life events	0.667	—	—	0.134	—	—
Use of neuraxial labor analgesia	0.063	—	—	0.068	—	—
Artificial membrane rupture during labor	0.102	—	—	0.126	—	—
**Mode of delivery**						
Spontaneous delivery	Reference	—	—	Reference	—	—
Forceps delivery	0.511	—	—	0.231	—	—
Cesarean delivery	0.617	—	—	0.227	—	—
Infant of male sex	0.189	—	—	0.126	—	—
2-year social support rating scale (score)	0.041	—	—	0.736	—	—
2-year Edinburgh postnatal depression scale (score)	<0.001	1.15 (1.08–1.22)	<0.001	0.019	1.09 (1.01–1.18)	0.037

P values in bold indicate < 0.05.

a Defined as Mental Development Index < 90.

b Factors with P < 0.15 in univariate analyses or considered clinically relevant were included. Multivariate logistic regression analysis was performed using a backward procedure. Hosmer-Lemeshow test of goodness of fit of the model was χ2 = 4.913, df = 6, P = 0.555.

c Factors with P < 0.15 in univariate analyses or considered clinically relevant were included. Multivariate logistic regression analysis was performed using a backward procedure. Hosmer-Lemeshow test of goodness of fit of the model was χ2 = 5.838, df = 8, P = 0.665.

## Discussion

In this prospective longitudinal study, we found that in offspring born to nulliparous women with single cephalic term pregnancy and planned vaginal delivery, 11.4% had below-average mental development at 2 years of age. Maternal depression at 2 years was associated with an increased risk of below-average mental development in their 2-year-old offspring, whereas maternal exposure to neuraxial labor analgesia was not.

Neuraxial analgesia is recognized as the most effective method to relieve labor pain (4). However, despite well-established benefits, concerns exist regarding the potential impact on the outcomes of offspring. Available studies suggest that neuraxial labor analgesia may produce both favorable and unfavorable effects on neonates and children, but evidences are still lacking (32). The fear of the potential unfavorable effects might have impeded some parturients and even health care professionals from accepting neuraxial labor analgesia, especially in China (33). Further studies on this topic will help mothers and professionals to consider neuraxial labor analgesia from a more rational perspective.

Low-concentration local anesthetic and opioid combinations are currently a common practice used for neuraxial labor analgesia, in order to provide effective analgesia while minimizing potential unfavorable effects (34). Over decades, concerns exist regarding the potential influence of labor analgesia on infant brain development (35). In the participating centers of the present study, parturients who did not request neuraxial analgesia were rarely given pharmacological analgesia. Therefore, it is not proper to perform a randomized controlled trial to explore the long-term effects of maternal exposure to epidural analgesia during labor. We therefore performed this observational follow-up study. We collected various sociodemographic and baseline data of parents and performed propensity score matching, in order to balance the effects of potential confounding factors due to the non-random exposure.

Neurodevelopment of children is a complex process and affected by multiple factors (36). However, due to lack of evidences, the long-term effects of neuraxial analgesia on offspring development remain unclear. A few human studies investigated the association between maternal exposure to epidural labor analgesia and offspring risk of autism spectrum disorders and reported heterogenous results (37, 38). In a study of rhesus monkeys, Golub and colleagues found that epidurally administered bupivacaine did not produce neonatal abnormalities or specific cognitive deficits, but altered the normal course of behavioral development (39). In a population-based cohort study, Randall and colleagues revealed that the use of neuraxial analgesia during labor and vaginal delivery was not associated with the presence of learning disabilities before the age of 19 years (40). In the present study, we did not find significant association between maternal exposure to neuraxial labor analgesia and offspring mental development outcome at 2 years of age; this is consistent with previous reports.

As the most important and closest person during early life stages, mothers play a critical role in the growth and development of children. Perinatal mental disorders of mothers may produce harmful effects on the risk of psychological and developmental disturbances in their offspring (8). Indeed, studies showed that infants of mothers with depression and personality disorder had higher levels of dysregulated behavior at 18 months (41), and children whose mothers had postnatal depression had more intellectual problems at 11 years of age (42). In a prospective longitudinal study, Sutter-Dallay and colleagues reported that maternal depression at 6 weeks was associated with poor cognitive performance of children at the age of 2 years, and part of this association could be attributed to chronic depressive symptoms (43). As a matter of fact, multiple studies revealed that persistent maternal depression is associated with a higher risk of negative outcomes in children (8, 44). In line with these, our results also confirmed that a higher maternal depressive score at 2 years was significantly associated with an increased risk of below-average mental development in the offspring. We cannot exclude the possibility that maternal depressive mood was a consequence of poor neurodevelopment of their children. However, this is less likely to be the case in our patients because both physical and psychomotor developments were similar in children of the two groups, and even the difference of mental development was not clinically important.

Although controversial, several studies including ours showed that neuraxial labor analgesia is associated with a decreased risk of postpartum depression (45, 46). Our underlying study found that neuraxial labor analgesia is also associated with a reduced risk of maternal depression at 2 years after childbirth (17). In our results of both the full and the matched cohort, the proportion with below-average MDI was slightly lower in children whose mothers received neuraxial analgesia during labor, but the difference was not statistically significant. It is possible that neuraxial labor analgesia may produce favorable effects on children's mental development by relieving early and late maternal depressive symptoms after childbirth. However, sample size of the present study was too small to reveal this effect and further studies are needed to test this hypothesis.

Despite strengths including a prospective design and use of propensity score matching to balance baseline variables, our study has some limitations. Firstly, this was a predefined secondary analysis of a 2-year longitudinal study and the sample size was not calculated for the current primary endpoint. Secondly, the distributions of MDI and PDI scores in the present study were skewed, indicating potential sampling or information biases. This is likely due to the limited sample size and the metropolitan medical centers participated in the study, which also limited the generalizability of our results. Besides, as an observational cohort study, we cannot establish the causal relationship between exposure to neuraxial labor analgesia and 2-year outcomes. Further studies with larger sample sizes and more participating centers are required to verify the findings.

## Conclusions

Our study did not find a significant association between maternal exposure to neuraxial labor analgesia and the risk of below-average mental developmental in 2-year-old children. High maternal depression score at 2 years was associated with an increased risk of below-average mental development in children. Further studies are warranted to clarify the effect of maternal neuraxial analgesia exposure on offspring neurocognitive outcomes.

## Data availability statement

The raw data supporting the conclusions of this article will be made available by the authors, without undue reservation.

## Ethics statement

The studies involving human participants were reviewed and approved by the Ethics Committee of Peking University First Hospital. Written informed consent to participate in this study was provided by the participants' legal guardian/next of kin.

## Author contributions

C-MD, TD, M-JX, LW, and D-XW conceived and designed the study. C-MD, TD, Z-HL, and S-TH collected data. C-MD, TD, Z-HL, J-HM, ML, W-LL, X-YL, DM, and D-XW analyzed and interpreted data. C-MD, TD, and Z-HL drafted the manuscript. D-XW critically revised the manuscript. All authors contributed to the article and approved the submitted version.

## Funding

This study was funded by the Interdisciplinary Clinical Research Project of Peking University First Hospital (2019CR36) and the National High Level Hospital Clinical Research Funding (Multi-center Clinical Research Project of Peking University First Hospital) (2022CR56). The funders had no roles in the study design, data collection, data analysis, data interpretation, report writing, or decision to submit.

## Conflict of interest

The authors declare that the research was conducted in the absence of any commercial or financial relationships that could be construed as a potential conflict of interest.

## Publisher's note

All claims expressed in this article are solely those of the authors and do not necessarily represent those of their affiliated organizations, or those of the publisher, the editors and the reviewers. Any product that may be evaluated in this article, or claim that may be made by its manufacturer, is not guaranteed or endorsed by the publisher.
