# Supplementary material for: Impact of maternal neuraxial labor analgesia exposure on offspring's neurodevelopment: A longitudinal prospective cohort study with propensity score matching
Source: Front Public Health. 2022 Jul 29;10:831538. doi: 10.3389/fpubh.2022.831538 (PMC9373030; doi:10.3389/fpubh.2022.831538)
Supplement: Supplementary file 1 [file Data_Sheet_1.docx]

Supplementary Tables

**Table S1. Demographic and baseline data of parturients who completed the 2-year follow-up and those who did not.**

|  | Completed follow-up (n=508) | Lost to follow-up (n=69) | ASD |
| --- | --- | --- | --- |
| Age at delivery (year) | 30 (28-32) | 30 (28-32) | 0.112 |
| Antenatal body mass index (kg/m^2^) | 27.3±2.9 | 27.3±2.4 | 0.025 |
| Maternal education >12 years | 486 (95.7%) | 67 (97.1%) | 0.070 |
| Covered by social health insurance | 480 (94.5%) | 64 (92.8%) | 0.076 |
| Smoking/drinking during pregnancy | 4 (0.8%) | 0 (0%) | 0.089 |
| History of surgery | 76 (15.0%) | 8 (11.6%) | 0.094 |
| Stressful life events ^a^ | 52 (10.2%) | 5 (7.2%) | 0.099 |
| Pregestational medical comorbidity ^b^ | 37 (7.3%) | 6 (8.7%) | 0.054 |
| Pregestational gynecological disease ^c^ | 51 (10.0%) | 5 (7.2%) | 0.093 |
| Pregnancy with obstetric disease ^d^ | 159 (31.3%) | 20 (29.0%) | 0.050 |
| Adverse pregnancy history ^e^ | 171 (33.7%) | 23 (33.3%) | 0.007 |
| Received neuraxial labor analgesia | 368 (72.4%) | 50 (72.5%) | 0.001 |

Data are mean ± SD, n (%) or median (interquartile range). ASD, absolute standardized difference (an ASD of ≥0.128 is considered imbalanced between the two groups).

^a^ Include bereavement, accidental injury, layoff or unemployment.

^b^ Include asthma, arrhythmia, latent glomerulonephritis, abnormal liver function and positive hepatitis B surface antigen.

^c^ Include hysteromyoma, ovarian cysts, dysfunctional uterine bleeding, polycystic ovary syndrome and pelvic inflammatory disease.

^d^ Include Impaired glucose tolerance, gestational diabetes mellitus, pregnancy-induced hypertension syndrome, preeclampsia and low free triiodothyronine and free thyroxine during pregnancy.

^e^ Include arrest of fetal development, spontaneous abortion, and induced abortion.

**Table S2. Descriptive statistics of Mental Development Index and Psychomotor Development Index scores by group**

| Variable | Total (n=508) | Full cohort | | | Matched cohort | | |
| --- | --- | --- | --- | --- | --- | --- | --- |
|  |  | Neuraxial analgesia (n=368) | No neuraxial analgesia (n=140) | *P* value | Neuraxial analgesia (n=254) | No neuraxial analgesia (n=133) | *P* value |
| Mental Development Index (level) ^a^ | 5 (4-5) | 5 (4-5) | 5 (4-5) | 0.050 | 5 (4-5) | 5 (4-5) | 0.051 |
| Mental development level |  |  |  |  |  |  |  |
| <70 | 16 (3.1%) | 10 (2.7%) | 6 (4.3%) |  | 7 (2.8%) | 5 (3.8%) |  |
| 70-79 | 9 (1.8%) | 6 (1.6%) | 3 (2.1%) |  | 3 (1.2%) | 2 (1.5%) |  |
| 80-89 | 33 (6.5%) | 20 (5.4%) | 13 (9.3%) |  | 11 (4.3%) | 12 (9.0%) |  |
| 90-109 | 162 (31.9%) | 116 (31.5%) | 46 (32.9%) |  | 77 (30.3%) | 43 (32.3%) |  |
| 110-119 | 233 (45.9%) | 172 (46.7%) | 61 (43.6%) |  | 124 (48.8%) | 60 (45.1%) |  |
| 120-129 | 50 (9.8%) | 40 (10.9%) | 10 (7.1%) |  | 29 (11.4%) | 10 (7.5%) |  |
| >130 | 5 (1.0%) | 4 (1.1%) | 1 (0.7%) |  | 3 (1.2%) | 1 (0.8%) |  |
| Psychomotor Development Index (level) ^a^ | 4 (4-5) | 4 (4-5) | 4 (4-5) | 0.708 | 4 (4-5) | 4 (4-5) | 0.943 |
| Psychomotor development level |  |  |  |  |  |  |  |
| <70 | 2 (0.4%) | 2 (0.5%) | 0 (0.0%) |  | 2 (0.8%) | 0 (0.0%) |  |
| 70-79 | 7 (1.4%) | 5 (1.4%) | 2 (1.4%) |  | 5 (2.0%) | 2 (1.5%) |  |
| 80-89 | 9 (1.8%) | 6 (1.6%) | 3 (2.1%) |  | 4 (1.6%) | 3 (1.5%) |  |
| 90-109 | 263 (51.8%) | 193 (52.4%) | 70 (50.0%) |  | 124 (48.8%) | 68 (51.1%) |  |
| 110-119 | 206 (40.6%) | 146 (39.7%) | 60 (42.9%) |  | 108 (42.5%) | 56 (42.1%) |  |
| 120-129 | 8 (1.6%) | 7 (1.9%) | 1 (0.7%) |  | 5 (2.0%) | 1 (0.8%) |  |
| >130 | 13 (2.6%) | 9 (2.4%) | 4 (2.9%) |  | 6 (2.4%) | 4 (3.0%) |  |

Data are presented as n (%) or median (interquartile range).

^a^ Classified into seven levels according to the Mental or Psychomotor Development Index scores, i.e., level 1: developmental delay (<70), level 2: borderline (70-79), level 3: below average (80-89), level 4: middle level (90-109), level 5: above average (110-119), level 6: good (120-129), and level 7: outstanding (≥130).

**Table S3. Univariate analyses of all factors in association with below average mental development in 2-year-old children ^a^**

| Factors | Full cohort (n=508) | | | Matched cohort (n=387) | | |
| --- | --- | --- | --- | --- | --- | --- |
|  | Number | Odds ratio (95% CI) | *P* value | Number | Odds ratio (95% CI) | *P* value |
| Baseline maternal data |  |  |  |  |  |  |
| Age at childbirth (year) | 508 | 1.07 (0.96-1.19) | 0.209 | 387 | 1.09 (0.96-1.24) | 0.210 |
| Antenatal body mass index (kg/m^2^) | 508 | 0.98 (0.89-1.08) | 0.704 | 387 | 1.02 (0.91-1.14) | 0.701 |
| Han nationality ^b^ | 479 | 1.26 (0.42-3.76) | 0.679 | 366 | 0.91 (0.20-4.05) | 0.900 |
| With religion ^c^ | 25 | 1.51 (0.50-4.57) | 0.463 | 20 | 1.57 (0.44-5.62) | 0.485 |
| Education >12 years | 486 | 0.81 (0.23-2.82) | 0.738 | 375 | 0.56 (0.12-2.67) | 0.470 |
| Without stable occupation | 23 | 0.34 (0.05-2.58) | 0.298 | 18 | 0.50 (0.06-3.84) | 0.503 |
| Without social health insurance | 28 | 0.58 (0.14-2.52) | 0.470 | 8 | --- | 0.999 |
| Total family income (each level increase) | 508 | 0.98 (0.67-1.46) | 0.932 | 387 | 0.92 (0.57-1.48) | 0.717 |
| History before last pregnancy |  |  |  |  |  |  |
| Premenstrual syndrome ^d^ | 49 | 0.48 (0.14-1.59) | 0.230 | 34 | 0.24 (0.03-1.83) | 0.170 |
| Medical comorbidity ^e^ | 37 | 0.94 (0.32-2.74) | 0.904 | 28 | 0.65 (0.15-2.85) | 0.567 |
| Gynecological disease ^f^ | 51 | 0.83 (0.32-2.18) | 0.703 | 42 | 0.90 (0.31-2.68) | 0.855 |
| Adverse pregnancy history ^g^ | 171 | 1.04 (0.59-1.85) | 0.888 | 147 | 0.98 (0.50-1.92) | 0.947 |
| Previous surgery | 76 | 0.90 (0.41-1.98) | 0.791 | 52 | 0.49 (0.15-1.66) | 0.254 |
| History of last pregnancy |  |  |  |  |  |  |
| Duration of pregnancy (day) | 508 | 1.02 (0.98-1.06) | 0.299 | 387 | 1.04 (0.99-1.09) | 0.172 |
| Smoking or alcohol drinking | 4 | --- | 0.999 | 1 | --- | >0.999 |
| Stressful life events ^h^ | 52 | 0.81 (0.31-2.12) | 0.667 | 38 | 0.22 (0.03-1.61) | 0.134 |
| Attend childbirth classes | 40 | 0.92 (0.47-1.81) | 0.806 | 300 | 1.00 (0.46-2.19) | 0.998 |
| Obstetric disease ^i^ | 159 | 0.99 (0.55-1.78) | 0.963 | 122 | 0.92 (0.45-1.88) | 0.827 |
| Prepartum hemoglobin (g/L) | 508 | 1.03 (0.82-1.30) | 0.793 | 387 | 1.04 (0.79-1.38) | 0.773 |
| Antenatal assessments (score) |  |  |  |  |  |  |
| Edinburgh Postnatal Depression Scale (score) | 508 | 1.04 (0.93-1.15) | 0.528 | 387 | 1.00 (0.88-1.13) | 0.944 |
| ENRICH Marital Satisfaction Scale (score) | 508 | 0.97 (0.88-1.07) | 0.478 | 387 | 0.96 (0.85-1.07) | 0.425 |
| Zung Self-Rating Anxiety Scale (score) | 508 | 1.03 (0.98-1.08) | 0.302 | 387 | 1.04 (0.98-1.11) | 0.206 |
| Social Support Rating Scale (score) | 508 | 1.06 (0.99-1.14) | 0.118 | 387 | 1.06 (0.97-1.15) | 0.212 |
| Baseline paternal data |  |  |  |  |  |  |
| Education of husband >12 years | 488 | 0.50 (0.16-1.54) | 0.227 | 374 | 0.62 (0.13-2.91) | 0.547 |
| Husband without stable occupation | 4 | --- | 0.999 | 3 | --- | 0.999 |
| Intrapartum maternal data |  |  |  |  |  |  |
| Use of neuraxial analgesia | 368 | 0.58 (0.33-1.03) | 0.063 | 254 | 0.54 (0.28-1.05) | 0.068 |
| Duration of neuraxial analgesia |  |  |  |  |  |  |
| No neuraxial analgesia | 140 | Reference |  | 133 | Reference |  |
| <4 h | 88 | 0.69 (0.31-1.53) | 0.359 | 62 | 0.53 (0.19-1.48) | 0.224 |
| 4-8 h | 139 | 0.54 (0.18-1.66) | 0.285 | 92 | 0.49 (0.20-1.23) | 0.129 |
| >8 h | 141 | 0.54 (0.17-1.64) | 0.274 | 100 | 0.59 (0.26-1.37) | 0.223 |
| Highest temperature during labor ≥37.5°C | 65 | 0.93 (0.40-2.14) | 0.860 | 47 | 0.79 (0.27-2.32) | 0.662 |
| Highest temperature during labor ≥38.0°C | 10 | 0.86 (0.11-6.91) | 0.887 | 7 | --- | 0.999 |
| Use of oxytocin | 344 | 0.75 (0.43-1.33) | 0.330 | 268 | 0.91 (0.45-1.84) | 0.800 |
| Artificial membrane rupture | 195 | 1.58 (0.91-2.74) | 0.102 | 150 | 1.67 (0.87-3.22) | 0.126 |
| Duration of first stage (min) | 385 | 0.97 (0.91-1.03) | 0.300 | 289 | 0.98 (0.91-1.06) | 0.604 |
| Duration of second stage (min) | 385 | 0.99 (0.99-1.00) | 0.205 | 289 | 0.99 (0.98-1.00) | 0.169 |
| Delivery mode |  |  |  |  |  |  |
| Spontaneous delivery | 336 | Reference |  | 256 | Reference |  |
| Forceps delivery | 49 | 0.70 (0.24-2.05) | 0.511 | 33 | 0.29 (0.04, 2.20) | 0.231 |
| Cesarean delivery | 123 | 1.17 (0.63-2.19) | 0.617 | 98 | 1.54 (0.77, 3.10) | 0.227 |
| Neonatal data |  |  |  |  |  |  |
| Male sex | 274 | 1.46 (0.83-2.56) | 0.189 | 207 | 1.70 (0.86-3.37) | 0.126 |
| Birth weight (100 g) | 508 | 1.01 (0.94-1.08) | 0.885 | 387 | 1.03 (0.95 -1.11) | 0.488 |
| 1-minute Apgar score <10 | 22 | 0.36 (0.05-2.72) | 0.321 | 17 | 0.53 (0.07-4.11) | 0.544 |
| 5-minute Apgar score <10 | 6 | --- | 0.999 | 5 | --- | 0.999 |
| Admission to neonatal ward ^j^ | 48 | 0.89 (0.34-2.35) | 0.819 | 40 | 0.68 (0.20-2.31) | 0.536 |
| 6-week postpartum data |  |  |  |  |  |  |
| Exclusive breast feeding | 351 | 0.91 (0.51-1.63) | 0.746 | 266 | 1.07 (0.52-2.18) | 0.855 |
| Persistent pain | 117 | 1.19 (0.64-2.23) | 0.587 | 88 | 1.15 (0.54-2.46) | 0.719 |
| Edinburgh Postnatal Depression Scale (score) | 508 | 1.05 (0.98-1.13) | 0.196 | 387 | 1.04 (0.96-1.14) | 0.350 |
| Postpartum depression ^k^ | 90 | 1.94 (1.03-3.63) | **0.039** | 67 | 1.45 (0.65-3.20) | 0.362 |
| 2-year maternal data |  |  |  |  |  |  |
| Body mass index at 2 years (kg/m^2^) | 508 | 0.95 (0.85-1.06) | 0.325 | 387 | 0.96 (0.84-1.10) | 0.546 |
| Maternal disease within 2 years ^l^ | 37 | 1.56 (0.62-3.92) | 0.344 | 24 | 0.78 (0.18-3.44) | 0.740 |
| Maternal surgery within 2 years ^m^ | 30 | 1.60 (0.59-4.37) | 0.355 | 21 | 1.48 (0.42-5.27) | 0.543 |
| Duration of breast-feeding (month) | 508 | 0.97 (0.94-1.01) | 0.183 | 387 | 0.98 (0.93-1.03) | 0.369 |
| Another childbirth | 19 | 1.48 (0.42-5.24) | 0.544 | 14 | 2.48 (0.66-9.28) | 0.178 |
| Social Support Rating Scale (score) | 508 | 0.94 (0.89-1.00) | **0.041** | 387 | 0.99 (0.92-1.06) | 0.736 |
| Edinburgh Postnatal Depression Scale (score) | 508 | 1.15 (1.08-1.22) | **<0.001** | 387 | 1.10 (1.02-1.18) | **0.019** |
| 2-year depression ^k^ | 46 | 4.16 (2.06-8.38) | **<0.001** | 31 | 2.86 (1.14 -7.13) | **0.025** |
| 2-year infant data |  |  |  |  |  |  |
| Age (month) | 508 | 0.71 (0.38-1.33) | 0.282 | 387 | 0.63 (0.26-1.19) | 0.214 |
| Height (cm) | 508 | 1.07 (0.98-1.18) | 0.264 | 387 | 1.16 (1.04-1.29) | 0.210 |
| Weight (kg) | 508 | 0.99 (0.82-1.20) | 0.897 | 387 | 1.07 (0.86-1.33) | 0.545 |
| Complementary feeding (month) | 508 | 0.95 (0.69-1.32) | 0.765 | 387 | 0.88 (0.60-1.29) | 0.507 |
| Physical development delay ^n^ | 8 | 1.11 (0.13-9.19) | 0.923 | 7 | 1.46 (0.17-12.42) | 0.731 |
| Pediatric disease within 2 years ^o^ | 52 | 1.74 (0.80-3.78) | 0.163 | 32 | 1.69 (0.61-4.68) | 0.310 |
| Pediatric surgery within 2 years ^p^ | 2 | --- | 0.999 | 2 | --- | 0.999 |

*P* values in bold indicate <0.05.

^a^ Defined as Mental Development Index <90.

^b^ Other nationalities include Manchu, Mongol, Huis, Koreans, and Yi.

^c^ Include Buddhism, Islam and Christianism.

^d^ Refers to symptoms of irritability, fatigue, depression and headache that repeatedly occurred during the luteal phase of the menstrual cycle and affected daily life. Diagnosis was confirmed by the gynecologists.

^e^ Include asthma, arrhythmia, thyroid disease, nephritis, nephritic syndrome, and positive hepatitis B surface antigen.

^f^ Include uterine fibroid, ovarian cyst, endometriosis, polycystic ovary syndrome and primary amenorrhea.

^g^ Include arrest of fetal development, spontaneous abortion and induced abortion.

^h^ Include bereavement, accidental injury, layoff or unemployment.

^i^ Includes gestational diabetes mellitus, pregnancy-induced hypertension and Hypothyroidism.

^j^ Neonates were admitted to neonatal ward because of fetal distress/asphyxia, aspiration pneumonia, premature birth/low-birth weight, glucopenia, jaundice/hyperbilirubinemia, infection, convulsion and anal atresia.

^k^ Defined as Edinburgh Postnatal Depression Scale ≥10.

^l^ Refers to new-onset diseases that occurred during the 2-year period after childbirth and required therapy, including mammitis/mammary abscess, pelvic floor dysfunction, polycystic ovary syndrome, hypothyroidism, hyperthyroidism, Hashimoto’s thyroiditis, thyroid cancer, cerebral infarction, IgA nephropathy, lumbar disc herniation, scoliosis and phalangeal fracture.

^m^ Refers to any surgical procedures performed during the 2-year period after childbirth, including second Caesarean delivery, induced abortion, vaginal polypectomy, hysteromyomectomy, adnexectomy, incision and drainage of mammary abscess, cholecystectomy, thyroidectomy, and incision and internal fixation metatarsal fracture.

^n^ Defined as height or weight <-2 standard deviation according to the *Reference standard for growth and development of children under 7 years of age in China* (26).

^o^ Includes any congenital (atrial septal defect, anal atresia and urachal fistula) and/or acquired diseases (bronchiolitis, febrile convulsion, Kawasaki disease, infant rash, eczema, urticarial, allergic dermatitis, pneumonia, anemia, inguinal hernia, and enteritis) that required therapy during the 2-year period.

^p^ Includes any surgical procedures (inguinal herniorrhaphy and urachal fistula resection) performed during the 2-year period.

**Table S4. Associations between neuraxial labor analgesia exposure and below-average mental development in 2-year-old children ^a^**

|  | Full cohort (n=508) | | | Matched cohort (n=387) | | |
| --- | --- | --- | --- | --- | --- | --- |
|  | No. with below-average MDI/Total | Crude odds ratio (95% CI) | Adjusted odds ratio (95% CI) ^b^ | No. with below-average MDI/Total | Crude odds ratio (95% CI) | Adjusted odds ratio (95% CI) ^b^ |
| Neuraxial labor analgesia |  |  |  |  |  |  |
| No | 22/140 | Reference | Reference | 19/133 | Reference | Reference |
| Yes | 36/368 | 0.58 (0.33-1.03) | 0.64 (0.35-1.18) | 21/254 | 0.54 (0.28-1.05) | 0.55 (0.28-1.09) |
| Duration of neuraxial analgesia |  |  |  |  |  |  |
| No neuraxial analgesia | 22/140 | Reference | Reference | 19/133 | Reference | Reference |
| <4 h | 10/88 | 0.69 (0.31-1.53) | 0.71 (0.31-1.65) | 5/62 | 0.53 (0.19-1.48) | 0.47 (0.16-1.36) |
| 4-8 h | 11/139 | 0.54 (0.18-1.66) | 0.53 (0.24-1.19) | 7/92 | 0.49 (0.20-1.23) | 0.51 (0.20-1.30) |
| >8 h | 15/141 | 0.54 (0.17-1.64) | 0.70 (0.35-1.46) | 9/100 | 0.59 (0.26-1.37) | 0.65 (0.27-1.55) |

MDI, Mental Development Index.

^a^ Defined as Mental Development Index <90.

^b^ Covariates included antenatal stressful life events, artificial membrane rupture during labor, mode of delivery, infant of male sex, 2-year Social Support Rating Scale (score), and 2-year Edinburgh Postnatal Depression Scale (score).

**Table S5. Factors associated with cognitive development delay in 2-year-old infants ^a^**

| Variables | Full cohort (n=508) | | | | | Matched cohort (n=387) | | |
| --- | --- | --- | --- | --- | --- | --- | --- | --- |
|  | Univariate analysis | Multivariate analysis ^b^ | | | Univariate analysis | | Multivariate analysis ^c^ | |
|  | *P* value | Odds ratio (95% CI) | *P* value | *P* value | | | Odds ratio (95% CI) | *P* value |
| ENRICH Marital Satisfaction Scale (score) | **0.006** | 0.87 (0.78-0.99) | **0.027** | **0.007** | | | 0.84 (0.74-0.96) | **0.009** |
| Zung Self-Rating Anxiety Scale (score) | 0.064 | --- | --- | 0.061 | | | --- | --- |
| Use of neuraxial analgesia | 0.336 | --- | --- | 0.547 | | | --- | --- |
| Emergency Cesarean delivery | 0.354 | --- | --- | 0.132 | | | --- | --- |
| 2-year Social Support Rating Scale (score) | **0.001** | 0.91 (0.83-0.99) | **0.038** | 0.056 | | | --- | --- |
| 2-year Edinburgh Postnatal Depression Scale (score) | **<0.001** | 1.13 (1.04-1.23) | **0.003** | **0.017** | | | 1.12 (1.01-1.24) | **0.031** |
| Pediatric disease within 2 years | 0.108 | --- | --- | 0.165 | | | --- | --- |

*P* values in bold indicate those <0.05.

^a^ Defined as Mental Development Index <80.

^b^ Factors with *P*<0.15 in univariate analyses (excluding 6-week Edinburgh Postnatal Depression Scale score because of collinearity with 2-year Edinburgh Postnatal Depression Scale score) were included were included. Multivariate logistic regression analysis was performed using a backward procedure. Hosmer-Lemeshow test of goodness of fit of the model was χ^2^=3.722, df=8, P=0.881.

^c^ Factors with *P*<0.15 in univariate analyses (excluding 6-week Edinburgh Postnatal Depression Scale score because of collinearity with 2-year Edinburgh Postnatal Depression Scale score) were included. Multivariate logistic regression analysis was performed using a backward procedure. Hosmer-Lemeshow test of goodness of fit of the model was χ^2^=4.461, df=8, P=0.813.

**Supplementary Figures**


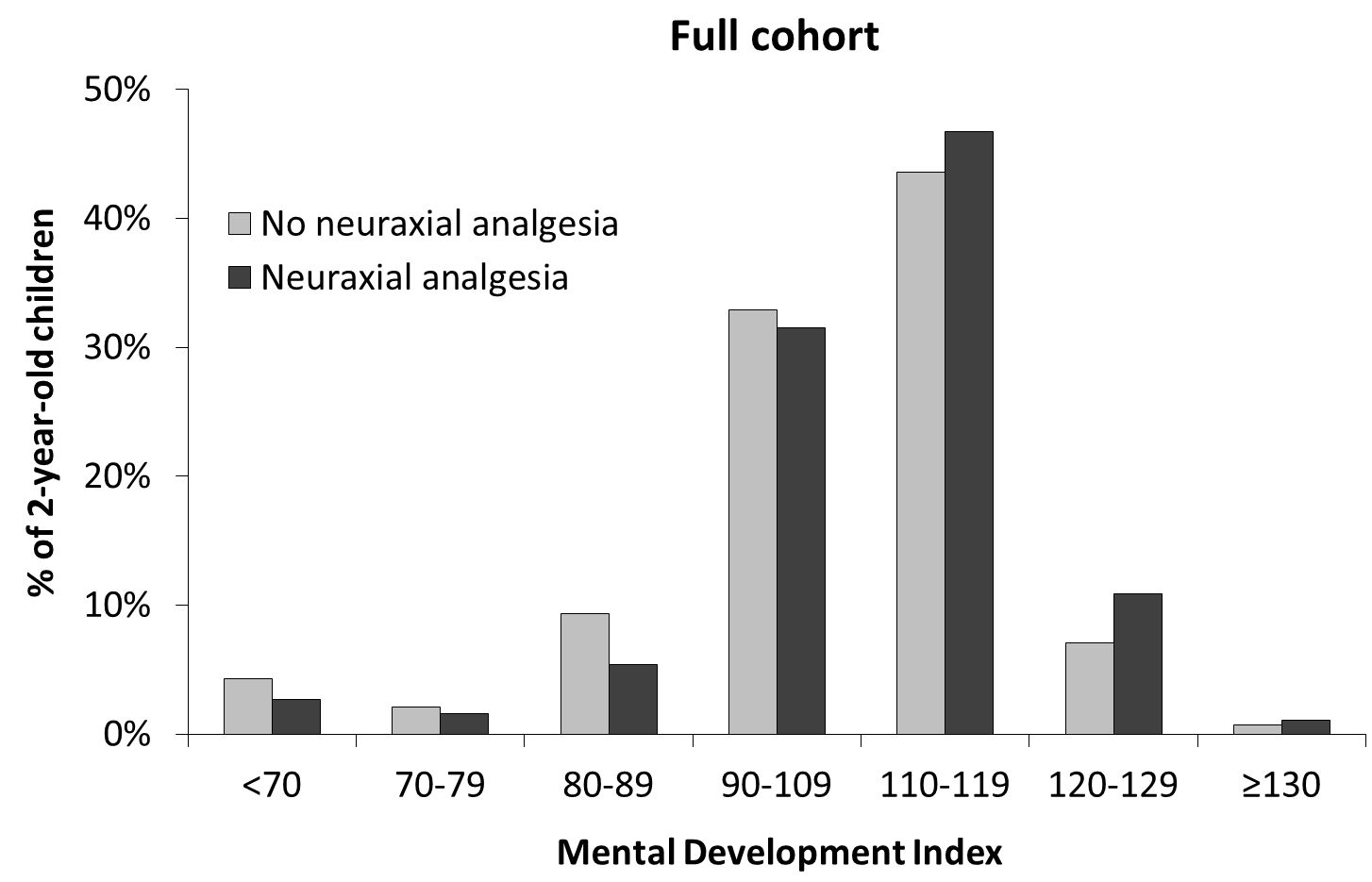


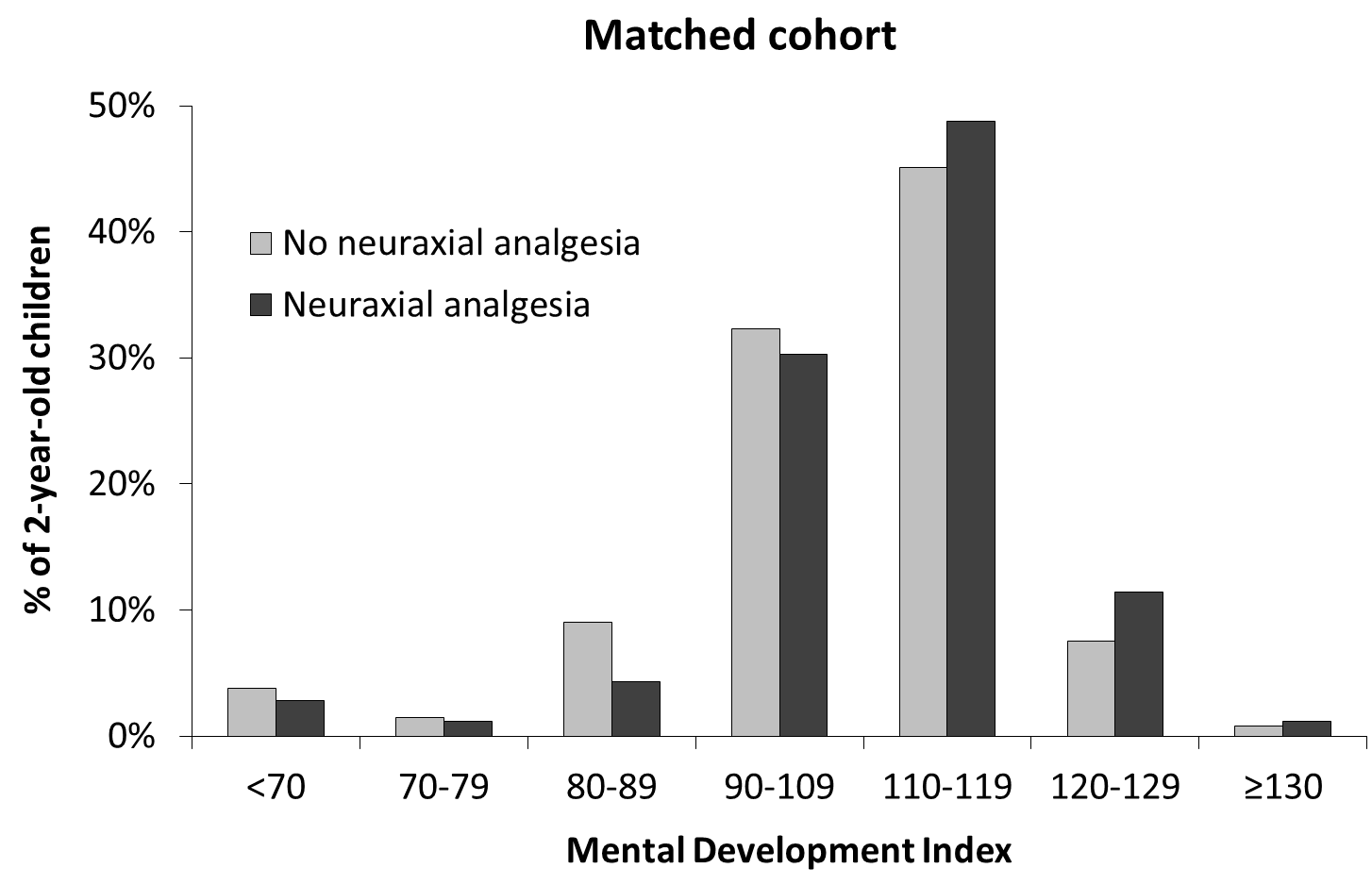


**Figure S1**. Column distribution of Mental Development Index before (full cohort) and after matching (matched cohort). The proportion with below-average Mental Development Index was slightly lower in children whose mothers received neuraxial analgesia but not statistically significantly (in full cohort: 9.8% [36/368] vs. 15.7% [22/140], *P*=0.060; in matched cohort: 8.3% [21/254] vs. 14.3% [19/133], *P*=0.065). Mental Development Index was classified into seven levels according to the score, i.e., developmental delay (<70), borderline (70-79), below average (80-89), middle level (90-109), above average (110-119), good (120-129), and outstanding (≥130).
